# Proposed Denosumab Biosimilar SB16 vs Reference Denosumab in Postmenopausal Osteoporosis: Phase 3 Results Up to Month 12

**DOI:** 10.1210/clinem/dgae611

**Published:** 2024-09-07

**Authors:** Bente Langdahl, Yoon-Sok Chung, Rafal Plebanski, Edward Czerwinski, Eva Dokoupilova, Jerzy Supronik, Jan Rosa, Andrzej Mydlak, Anna Rowińska-Osuch, Ki-Hyun Baek, Audrone Urboniene, Robert Mordaka, Sohui Ahn, Young Hee Rho, Jisuk Ban, Richard Eastell

**Affiliations:** Department of Endocrinology, Aarhus University Hospital and Department of Clinical Medicine, Aarhus University, Aarhus, 8200, Denmark; Department of Endocrinology and Metabolism, Ajou University School of Medicine, Suwon, 16499, Republic of Korea; Institute on Aging, Ajou University Medical Center, Suwon, 16499, Republic of Korea; Department of Clinic of Healthy Bone, Klinika Zdrowej Kosci, Lodz, 91-843, Poland; Clinical Trial Center, Krakowskie Centrum Medyczne, Krakow, 31-501, Poland; Department of Rheumatology, MEDICAL PLUS sro, Uherske Hradiste, 686 01, Czech Republic; Faculty of Pharmacy, Department of Pharmaceutical Technology, Masaryk University, Brno, 612 00, Czech Republic; Department of Rheumatology, OsteoMedic sc A Racewicz J Supronik, Bialystok, 15-351, Poland; Osteocenter, Affidea Praha, s.r.o., Praha, 148 00, Czech Republic; Medical Department, ETG Siedlce, Siedlce, 08-110, Poland; Department of Clinical Trials, ETG Warszawa, Warszawa, 02-677, Poland; Division of Endocrinology and Metabolism, Department of Internal Medicine, Yeouido St. Mary's Hospital, College of Medicine, The Catholic University of Korea, Seoul, 07345, Republic of Korea; Department of Family Medicine, JSC Saules seimos medicinos centras, Kaunas, 49449, Lithuania; Department of Santa Familia PTG Lodz, Santa Sp. z o.o., Lodz, 90-302, Poland; Department of Biometrics, Samsung Bioepis Co., Ltd., Incheon, 21987, Republic of Korea; Department of Clinical Development, Samsung Bioepis Co., Ltd., Incheon, 21987, Republic of Korea; Department of Clinical Development, Samsung Bioepis Co., Ltd., Incheon, 21987, Republic of Korea; Division of Clinical Medicine, University of Sheffield, Sheffield S10 2RX, UK

**Keywords:** menopause, metabolic bone disease, osteoporosis, clinical trials

## Abstract

**Context:**

SB16 is a proposed biosimilar to reference denosumab (DEN; brand name: Prolia).

**Objective:**

This phase 3 randomized, double-blind, multicenter study evaluated the biosimilarity of SB16 to DEN in women with postmenopausal osteoporosis (NCT04664959).

**Design:**

The study included 457 postmenopausal osteoporosis patients who had a lumbar spine or total hip T-score between −2.5 and −4. Patients were randomized in a 1:1 ratio to receive either 60 mg of SB16 or DEN subcutaneously at month 0 and month 6. At month 12, patients were rerandomized to continue with the assigned treatment or switch from DEN to SB16 up to month 18. This report includes results up to month 12.

**Methods:**

The primary endpoint was the percent change from baseline in lumbar spine bone mineral density (BMD) at month 12. Secondary endpoints including the percent change from baseline in BMD of the lumbar spine (except for month 12), total hip, and femoral neck; pharmacokinetic, pharmacodynamic (serum C-telopeptide of type I collagen, and procollagen type I N-terminal propeptide), safety, and immunogenicity profiles were measured up to month 12.

**Results:**

The least-squares mean differences in percent change from baseline in lumbar spine BMD at month 12 were 0.33% (90% CI, −0.25 to 0.91) in the full analysis set and 0.39% (95% CI, −0.36 to 1.13) in the per-protocol set; both within the predefined equivalence margin. The secondary endpoints were comparable between the 2 treatment groups.

**Conclusion:**

The reported efficacy, pharmacokinetic, pharmacodynamic, safety, and immunogenicity data support the biosimilarity of SB16 to DEN.

Postmenopausal osteoporosis (PMO) is a highly prevalent chronic bone disease that affects approximately 20% of women older than age 50 years in the United States and is characterized by decreased bone mass and quality and an increased risk of fragility fractures (1, 2). Various pharmacological treatments with different mechanisms of action have been approved for the treatment of PMO and prevention of PMO-related fractures (3, 4). Reference denosumab (brand name: Prolia [DEN]) is a human monoclonal antibody to the receptor activator of nuclear factor κB ligand and blocks the activation of its receptor, RANK. Impaired RANK signaling inhibits osteoclast development and activity, resulting in decreased bone resorption and a subsequent increase in bone density (5, 6). SB16 has been developed as a proposed biosimilar to DEN (7, 8). A biosimilar is a biological medicinal product that is highly similar to an already approved biologic (reference product) in terms of quality, efficacy, and safety. Similarity is established through comprehensive comparability studies to generate the “totality of the evidence” that demonstrates high similarity and clinical equivalence of the proposed biosimilar to the reference product (9, 10). Using state-of-the-art analytical methods, SB16 was shown to exhibit highly similar physicochemical, structural, and biologic properties as compared to DEN (data on file). In addition, SB16 has been shown to be equivalent to DEN with regard to pharmacokinetics (PK), pharmacodynamics (PD), safety, and immunogenicity in a phase 1 study in healthy volunteers (NCT04621318). The objective of this trial was to evaluate biosimilarity of SB16 to DEN in terms of efficacy, safety, PK, PD, and immunogenicity in patients with PMO. In this report, we describe the 12-month results of a phase 3 equivalence trial comparing SB16 and DEN in patients with PMO during the main period (month 0 to month 12), which was followed by a switching period from month 12 to month 18.

## Material and Methods

### Study Design

This was a phase 3 randomized, double-blind, parallel group, multicenter, equivalence study to evaluate the efficacy, safety, PK, PD, and immunogenicity of SB16 (Samsung Bioepis Co., Ltd., Incheon, Republic of Korea) and DEN (Prolia, Amgen, Thousand Oaks, CA, USA) in patients with PMO. The study was conducted at 40 sites in 5 countries (Czech Republic, Denmark, Lithuania, Poland, and Republic of Korea) and started on November 26, 2020. The study lasted approximately 25 months. Included patients were postmenopausal women aged 55 to 80 years with a bone mineral density (BMD) T-score of the lumbar spine or total hip between −2.5 and −4, who were treatment-naïve to biologic medicines (defined as any therapeutic monoclonal antibody or fusion receptor protein, including denosumab, denosumab biosimilars, or romosozumab) at screening, and who had at least 3 evaluable vertebrae from L1 to L4 and 1 evaluable hip joint for BMD measurements. Postmenopausal was defined as the absence of menstrual periods for at least 12 months before screening for no other pathological or physiological reason. The central imaging center confirmed the BMD T-score at screening and determined the number of evaluable sites for BMD measurements. To calculate the T-score of the hip, NHANES III reference ranges for the US population for both GE and Hologic devices were used (11). For the spine, published manufacturer's ranges for the US population served as reference range for the calculation of the T-score. Key exclusion criteria were: (1) 1 severe or more than 2 moderate vertebral fractures on spinal X-ray according to the Genant classification (12) (as determined by the central imaging center), (2) a history of hip fracture or bilateral hip replacement, (3) serum 25-hydroxy-(OH)-vitamin D levels <50 nmol/L (<20 ng/mL), (4) albumin-adjusted serum calcium levels <2.1 mmol/L (<8.4 mg/dL) or >2.62 mmol/L (>10.5 mg/dL), (5) use of oral bisphosphonates for the treatment of osteoporosis at any dose either for >3 years cumulatively at screening or ≤3 years cumulatively and stopped <1 year before screening, and (6) estimated glomerular filtration rate< 45 mL/min according to the Modification of Diet in Renal Disease formula or under dialysis.

### Study Procedures

The clinical study protocol and protocol amendments were reviewed and approved by an Independent Ethics Committee or institutional review board. This study was conducted in compliance with International Council for Harmonization and Good Clinical Practice guidelines and the Declaration of Helsinki. Informed consent was obtained from each patient before entering the study. Each patient was assigned a unique subject and randomization number by the Interactive Web Response System at screening and randomization, respectively, to ensure that treatment group allocation was unbiased and concealed from patients, investigators, and other study personnel. The study was registered at clinicaltrials.gov (NCT04664959).

Main period (month 0 to month 12): Upon screening, eligible patients were randomized in a 1:1 ratio to receive either SB16 or DEN, 60 mg subcutaneously, at month 0 and month 6. All patients received at least 1 g of elemental calcium and 800 IU of vitamin D daily during the main period. BMD measurements of the lumbar spine (L1-L4), total hip, and femoral neck were performed at screening, month 6, and month 12 using GE Lunar or Hologic dual energy X-ray absorptiometry machines that were certified by the central reading center. The use of dual energy X-ray absorptiometry machines from different manufacturers at follow-up timepoints was not allowed. The main period was followed by a switching period (month 12 to month 18). Results up to month 12 are presented here for all outcomes.

### Study Endpoints

The primary endpoint was the percent change from baseline in lumbar spine BMD at month 12. Secondary efficacy endpoints included the percent changes from baseline in (1) lumbar spine BMD at month 6, (2) total hip BMD at month 6 and month 12, and (3) femoral neck BMD at month 6 and month 12. Safety endpoints included the incidences of adverse events (AEs), serious AEs (SAEs), and AEs of special interest (AESIs) (ie, hypocalcemia, hypersensitivity to the study drug, osteonecrosis of the jaw, atypical femoral fractures, and skin infections). Serum drug concentration measurements were performed at all visits to compare the PK of SB16 and DEN. PD endpoints included serum concentration measurements of C-telopeptide of type I collagen (CTX) and procollagen type I N-terminal propeptide (P1NP) at all visits and area under the effect curve from timepoint 0 to month 6 (AUEC_0-M6_) of the percent change from baseline in serum CTX. The levels of serum CTX were measured by Elecsys β-CrossLaps Kit (Catalog # 11972308122, RRID: AB_2905599) and P1NP were measured by Elecsys total P1NP Kit (Catalog # 03141071190, RRID: AB_2782967) using electrochemiluminescence (COBAS 8000; Roche Diagnostics, Germany). The lower limits of quantification were 0.043 ng/mL for serum CTX and 9.92 ng/mL for serum P1NP. The incidence of antidrug antibodies (ADA) and neutralizing antibodies was measured at all visits to assess immunogenicity.

### Statistical Analysis

#### Sample size calculation

Equivalence margins for the mean percent change from baseline in lumbar spine BMD at month 12 were estimated based on available data for DEN (13-15). Based on an equivalence margin of [−1.45%, 1.45%], a sample size of 432 patients (216 patients per treatment group) was determined to demonstrate equivalence for the primary efficacy endpoint by providing 80% power to reject the null hypothesis at a significance level of 10%.

#### Analysis sets

The randomized set included all patients who received a randomization number. The Full Analysis Set (FAS) consisted of all randomized patients except for those who inadvertently had no lumbar spine BMD assessment result and did not receive any study drug after randomization. The Per-Protocol Set (PPS) included all patients from the FAS with a lumbar spine BMD assessment at baseline and month 12 and without major protocol deviations impacting lumbar spine BMD results. The Safety Set 1 (SAF1) consisted of all patients who had received at least 1 dose of the study drug. Patients in the SAF1 who had at least 1 CTX or P1NP measurement without any major protocol deviations impacting PD results were included in the PD Analysis Set. The PK Analysis Set included all patients in the SAF1 who had at least 1 drug concentration measurement.

#### Primary endpoint

The primary analysis sought to demonstrate equivalence of SB16 and DEN for the primary endpoint and percent change from baseline in lumbar spine BMD at month 12 in the FAS and PPS. For this, an analysis of covariance model of the primary endpoint with baseline BMD as covariate and treatment group as factor was used. Equivalence was met if the 90% CI of the mean difference in the primary endpoint between SB16 and DEN was contained within the predefined equivalence margin specified for the FAS (−1.45% to 1.45%) for US Food and Drug Administration purposes or if the 95% CI was within the predefined equivalence margin specified for the PPS (−2.0% to 2.0%) for European Medicines Agency purpose. For the primary analysis in the FAS, missing data were imputed using a multiple imputation method under the “missing at random” assumption. Analyses were performed using Statistical Analysis System (SAS) Software Version 9.3 (SAS Institute Inc.).

#### Secondary endpoints

The percent changes from baseline in lumbar spine BMD at month 6 and total hip BMD and femoral neck BMD up to month 12 were described by treatment group using summary statistics.

For the safety analyses, patients were analyzed according to the study treatment they had received. For all AE and SAE tables, patients were counted once per each preferred term and system organ class. All reported AEs were coded using MedDRA version 23.0. PK, PD, and immunogenicity results were summarized descriptively by treatment group. No hypothesis testing was done for the secondary endpoints.

## Results

### Patient Disposition

**Figure 1.**
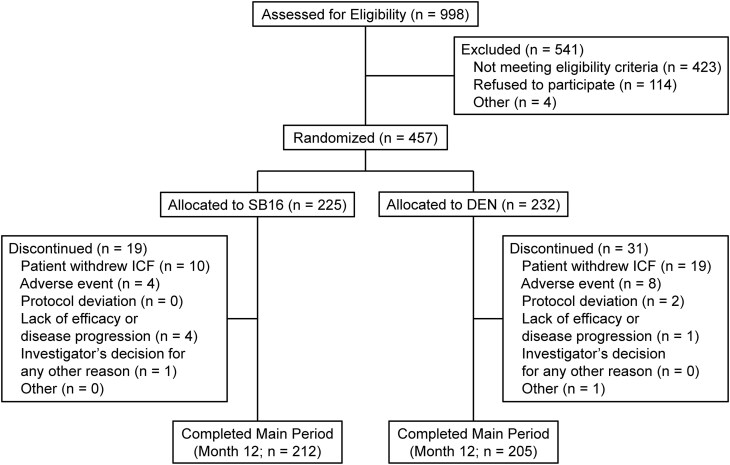
Patient disposition up to month 12. If a patient discontinued the study before rerandomization at month 12 but performed the BMD assessment at month 12 (eg, at an early termination visit), the patient was considered to have completed the main period. For this reason, 6 patients from the SB16 group and 4 patients from the DEN group were counted as completers despite discontinuation.

Abbreviations: BMD, bone mineral density; DEN, reference denosumab; ICF, informed consent form; SB16, proposed biosimilar to DEN.

Of 457 randomized patients, 456 (99.8%) received at least 1 dose of the study drug and 417 (91.2%) completed the main period (Fig. 1). The completion rate was comparable between the 2 treatment groups (SB16: n = 212 [94.2%] vs DEN: n = 205 [88.4%]). Overall, during the main period, 50 (10.9%) patients discontinued the study (SB16: n = 19 [8.4%] vs DEN: n = 31 [13.4%]). The most common primary reason for study discontinuation during the main period was withdrawal of consent (29 [6.3%] patients).

### Baseline Demographics and Disease Characteristics

Baseline demographics and disease characteristics were well-balanced between the 2 treatment groups (Table 1). Mean (SD) age was 66.5 (5.9) vs 66.3 (6.0) years in the SB16 and DEN group, respectively. Mean (SD) body mass index was 25.2 (3.8) kg/m^2^ in the SB16 group and 24.9 (3.5) kg/m^2^ in the DEN group. BMD T-scores at the lumbar spine were comparable between the SB16 and DEN group (SB16: −3.04 [0.47]; DEN: −3.05 [0.50]). Mean (SD) serum CTX was 0.44 (0.20) ng/mL in both groups. Other baseline demographic and disease characteristics were comparable between the SB16 and DEN groups.

**Table 1.** Baseline demographic and disease characteristics (RAN)

Characteristics	SB16 N = 225	DEN N = 232	Total N = 457
Age (y), mean (SD)	66.5 (5.9)	66.3 (6.0)	66.4 (5.9)
Age group, n (%)			
≥65 y	136 (60.4)	137 (59.1)	273 (59.7)
Race, n (%)			
Asian	18 (8.0)	23 (9.9)	41 (9.0)
White	207 (92.0)	208 (89.7)	415 (90.8)
Other	0 (0.0)	1 (0.4)	1 (0.2)
BMI (kg/m^2^), mean (SD)	25.2 (3.8)	24.9 (3.5)	25.0 (3.6)
BMI category, n (%)			
≥25 kg/m^2^	108 (48.0)	100 (43.1)	208 (45.5)
Years since menopause, mean (SD)	16 (7)	16 (8)	16 (8)
Prevalent vertebral fracture, n (%)			
Yes	104 (46.2)	117 (50.4)	221 (48.4)
No	119 (52.9)	113 (48.7)	232 (50.8)
Not assessable*^a^*	2 (0.9)	2 (0.9)	4 (0.9)
Serum 25-OH-vitamin D levels (nmol/L), mean (SD)	95.2 (40.5)	92.1 (34.8)	93.6 (37.7)
eGFR using MDRD equation (mL/min/SA), mean (SD)	79.8 (13.5)	80.6 (15.5)	80.2 (14.5)
Serum PTH (pmol/L), mean (SD)	4.2 (1.6)	4.1 (1.7)	4.2 (1.6)
Prior use of oral bisphosphonates, n (%)	42 (18.7)	33 (14.2)	75 (16.4)
T-score, mean (SD)			
Lumbar spine	−3.04 (0.47)	−3.05 (0.50)	−3.05 (0.48)
Total hip	−1.81 (0.77)	−1.82 (0.74)	−1.81 (0.76)
Femoral neck	−2.16 (0.61)	−2.16 (0.63)	−2.16 (0.62)
Serum CTX levels (ng/mL), mean (SD)	0.44 (0.20)	0.44 (0.20)	0.44 (0.20)

The years since menopause were calculated with the following formula: (randomization date — date of last menstruation + 1) divided by 365.25. Age was calculated by subtracting the birth year from the year in which informed consent was obtained.

Abbreviations: BMI, body mass index; CTX, C-telopeptide of type I collagen; DEN, reference denosumab; eGFR, estimated glomerular filtration rate; MDRD, modification of diet in renal disease; N, number of patients in the RAN in each treatment group; RAN, Randomized Set; SA, 1.73 m^2^; SB16, proposed biosimilar to DEN.

^*a*^At least 1 vertebra with unknown fracture status and no fracture at other evaluable vertebrae.

### Efficacy

The least-squares (LS) mean and SE percent change from baseline in lumbar spine BMD was 5.63% (0.25%) in the SB16 and 5.30% (0.25%) in the DEN group with a treatment difference of 0.33% (90% CI, −0.25% to 0.91%) in the FAS group (Table 2). In the PPS group, the respective changes were 5.71% (0.27%) in the SB16 and 5.32% (0.27%) in the DEN group with a treatment difference of 0.39% (95% CI, −0.36% to 1.13%). The 2-sided CI of the treatment difference between SB16 and DEN was within the prespecified equivalence margins in both the FAS and PPS.

**Table 2.** Analysis of the percent change from baseline in lumbar spine BMD at month 12 (FAS and PPS)

Analysis set	Treatment group	N	n	LSmean (SE)	Difference (SB16—DEN)		
					LSmean (SE)	90% CI	95% CI
FAS	SB16	225	225	5.63 (0.25)	0.33 (0.35)	−0.25 to 0.91	−0.36 to 1.03
	DEN	231	231	5.30 (0.25)			
PPS	SB16	191	191	5.71 (0.27)	0.39 (0.38)	−0.24 to 1.01	−0.36 to 1.13
	DEN	192	192	5.32 (0.27)			

Inferential statistics were based on an analysis of covariance model with baseline lumbar spine BMD as covariate and treatment group as fixed factor. For the FAS, missing data were imputed using a multiple imputation method under the “missing at random” assumption.

Abbreviations: BMD, bone mineral density; DEN, reference denosumab; FAS, Full Analysis Set; LSmean, least-squares mean; N, total number of patients in the FAS or PPS in each treatment group; n, number of patients with available data at month 12; PPS, Per-Protocol Set; SB16, proposed biosimilar to DEN.

**Figure 2.**
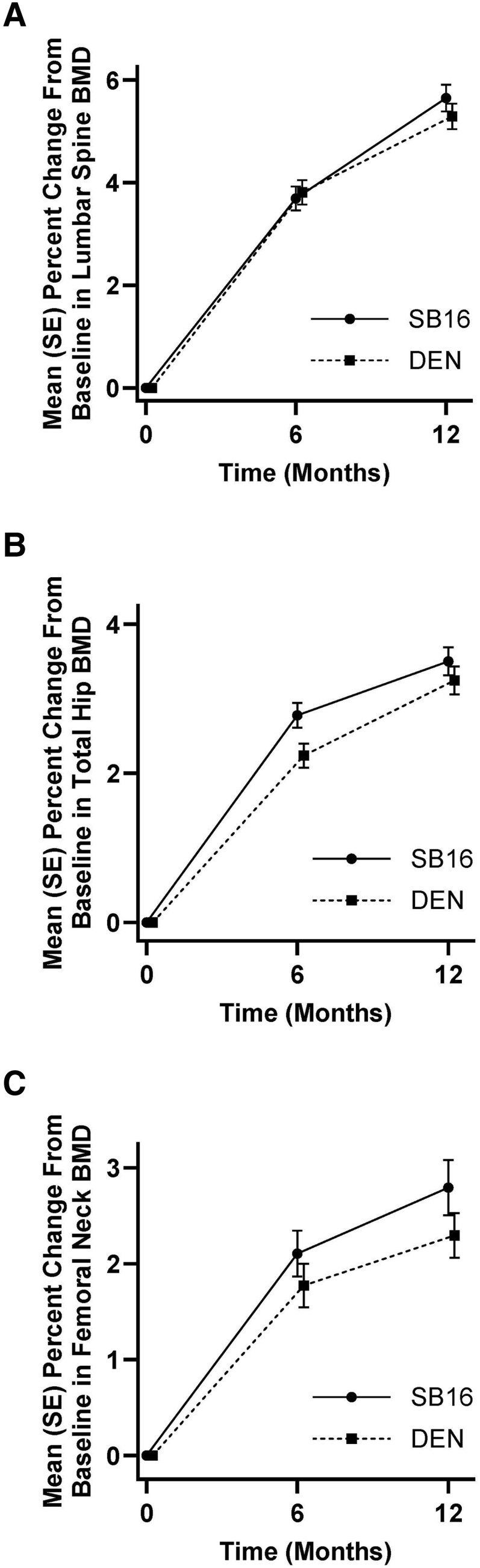
Changes in BMD up to month 12 (FAS). Mean (SE) percent changes from baseline in lumbar spine BMD (A), total hip BMD (B), and femoral neck BMD (C) up to month 12.

Abbreviations: BMD, bone mineral density; DEN, reference denosumab; FAS, Full Analysis Set; SB16, proposed biosimilar to DEN.

Mean percent changes from baseline in lumbar spine BMD at month 6 were 3.69% and 3.81% in the SB16 and DEN groups, respectively (Fig. 2A). Total hip BMD increased by 2.78% in the SB16 group and by 2.24% in the DEN group at month 6 and by 3.50% in the SB16 group and 3.25% in the DEN group at month 12 (Fig. 2B). At month 6, mean percent changes in femoral neck BMD were 2.11% in the SB16 group and 1.77% in the DEN group. At month 12, changes were 2.79% and 2.30% in the SB16 and DEN group, respectively (Fig. 2C).

### PD and PK

**Figure 3.**
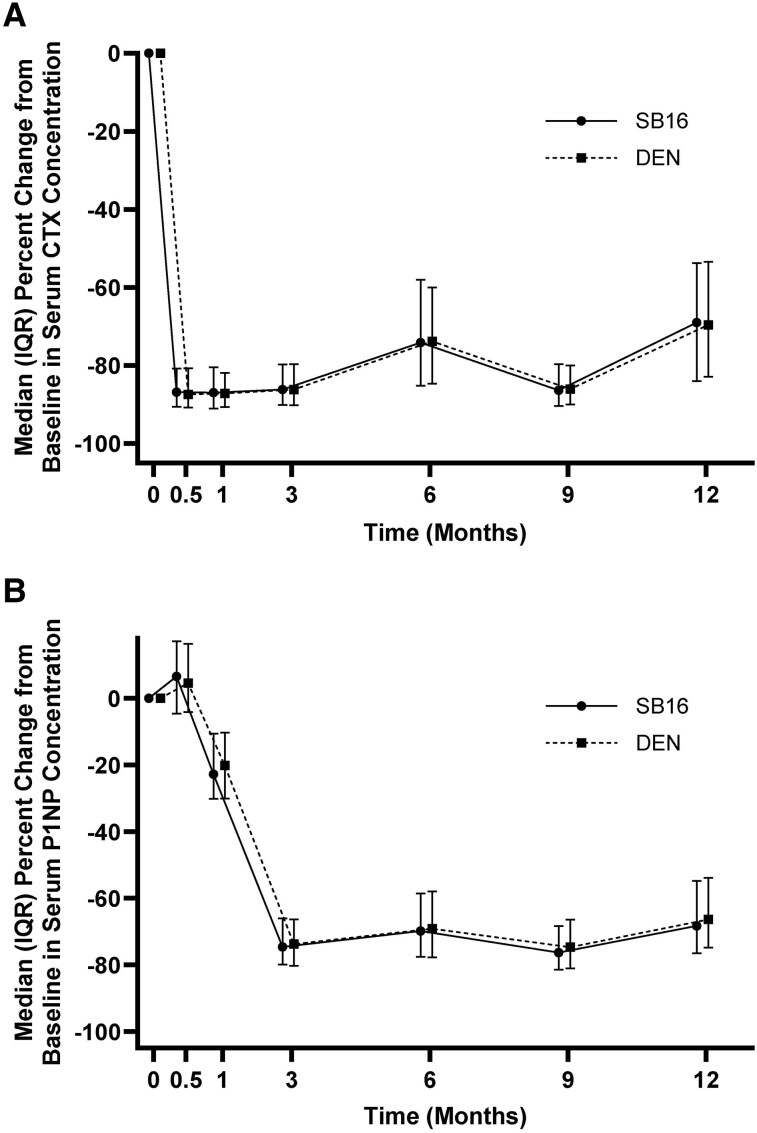
Changes in bone turnover marker concentrations up to month 12 (PDS). Median (IQR) percent changes from baseline in serum CTX (A) and serum P1NP (B) concentration profiles up to month 12.

Abbreviations: CTX, C-telopeptide of type I collagen; DEN, reference denosumab; IQR, interquartile range; P1NP, procollagen type I N-terminal propeptide; PDS, Pharmacodynamic Analysis Set; SB16, proposed biosimilar to DEN.

Median percent changes from baseline in serum CTX concentrations up to month 12 were comparable between the SB16 and DEN group until month 12 (Fig. 3A). At month 12, the median percent changes from baseline were 69.0% in the SB16 group and 69.6% in the DEN group. AUEC_0-M6_ of the percent change from baseline in serum CTX concentrations was comparable between the 2 treatment groups. The ratio of the geometric LS means of AUEC_0-M6_ in percent change from baseline in serum CTX concentration between the SB16 and DEN group was 0.98 (90% CI, 0.94-1.03). Serum P1NP concentrations decreased over time starting at month 1. Median percent changes from baseline in serum P1NP concentrations up to month 12 were comparable between the SB16 and DEN group (Fig. 3B). Mean serum concentrations of SB16 and DEN were comparable between the groups up to month 12.

### Safety and Immunogenicity

The mean duration of study drug exposure up to month 12 was 351.8 days in the SB16 group and 338.2 days in the DEN group. A total of 323 (70.8%) patients experienced at least 1 treatment-emergent AE (TEAE) during the main period (SB16: n = 159 [70.7%]; DEN: n = 164 [71.0%]; Table 3). The majority of TEAEs were mild or moderate in intensity and were not considered to be related to the study drug. A total of 55 (12.1%) patients experienced 61 treatment-emergent AESIs during the main period (SB16: n = 24 [10.7%]; DEN: n = 31 [13.4%]). The most frequently reported AESI was hypocalcemia and occurred in 49 (10.7%) patients overall with a comparable incidence in the SB16 (n = 22 [9.8%]) and DEN (n = 27 [11.7%]) group. Most events of hypocalcemia were grade 1 in severity according to the Common Terminology Criteria for Adverse Events version 5.0 (CTCAE v5.0). None of the events were associated with clinical manifestations such as tetany or seizures. No events of osteonecrosis of the jaw or atypical femoral fracture were reported during the main period. TEAEs of skeletal fractures were reported in 8 (3.6%) patients in the SB16 group and 1 (0.4%) patient in the DEN group during the main period. Three (1.3%) patients in the SB16 and 1 (0.4%) patient in the DEN group reported injection site reactions. A total of 16 (3.5%) patients (SB16: n = 8 [3.6%]; DEN: n = 8 [3.5%]) had 20 SAEs in the main period. Of these, 12 were severe, 7 were moderate, and 1 was mild. None of the SAEs was considered to be related to the study drug. Four (1.8%) patients in the SB16 group experienced TEAEs that led to permanent discontinuation of the study drug (arachnoid cyst, headache, acute phase reaction, tooth fracture, and alopecia) compared to 8 (3.5%) patients in the DEN group (presyncope, alopecia, dental caries, hemorrhoids, noninfective gingivitis, COVID-19, diverticulitis, upper respiratory tract infection, breast cancer, and lung adenocarcinoma). None of the TEAEs and SAEs had a fatal outcome. The incidence of positive ADA was <1.0% up to 12 months (data not shown). One (0.4%) patient in the SB16 and 2 (0.9%) patients in the DEN group were overall positive for ADA up to month 12. None of the 3 patients was neutralizing antibody positive.

**Table 3.** Safety profiles of SB16 and DEN up to month 12 (SAF1)

Safety events	SB16 N = 225	DEN N = 231
Patients with TEAEs, n (%)	159 (70.7)	164 (71.0)
TEAE severity, n (%)		
Mild	91 (40.4)	80 (34.6)
Moderate	62 (27.6)	78 (33.8)
Severe	6 (2.7)	6 (2.6)
Study drug-related TEAEs, n (%)	26 (11.6)	33 (14.3)
TEAEs of special interest, n (%)	24 (10.7)	31 (13.4)
Hypocalcemia	22 (9.8)	27 (11.7)
Hypersensitivity to the study drug	1 (0.4)	3 (1.3)
Skin infections	1 (0.4)	1 (0.4)
Osteonecrosis of the jaw	0 (0.0)	0 (0.0)
Atypical femoral fractures	0 (0.0)	0 (0.0)
Serious TEAEs, n (%)	8 (3.6)	8 (3.5)
Injection site reactions, n (%)	3 (1.3)	1 (0.4)
TEAEs leading to study drug discontinuation, n (%)	4 (1.8)	8 (3.5)
TEAEs leading to death, n (%)	0 (0.0)	0 (0.0)
Any TEAEs occurring in >5% of patients, n (%)	84 (37.3)	80 (34.6)
Hypocalcemia	22 (9.8)	27 (11.7)
Arthralgia	16 (7.1)	9 (3.9)
COVID-19	16 (7.1)	15 (6.5)
Headache	16 (7.1)	10 (4.3)
Urinary tract infection	12 (5.3)	5 (2.2)
Upper respiratory tract infection	11 (4.9)	12 (5.2)
Nasopharyngitis	10 (4.4)	14 (6.1)

In case a patient had multiple events with different severity (or causality) assessments, the patient was counted only once using the worst severity (or causality) assessment for the number of patients (n).

Abbreviations: DEN, reference denosumab; N, total number of patients in the SAF1 in each treatment group; n, number of patients with event; SAF1, Safety Analysis Set 1; SB16, proposed biosimilar to DEN; TEAE, treatment-emergent adverse event.

## Discussion

This randomized, double-blind, multicenter clinical study evaluated the biosimilarity of SB16 to DEN in terms of efficacy, safety, PK, PD, and immunogenicity in patients with PMO. The primary endpoint, mean percent change from baseline in lumbar spine BMD at month 12, was equivalent between the SB16 and DEN groups. As the 2-sided 90% or 95% CI of the treatment difference between SB16 and DEN was within the prespecified equivalence margins in both the FAS and PPS, equivalence in efficacy between the 2 groups was demonstrated. Change in lumbar spine BMD was chosen as a primary endpoint because previous trials with DEN have revealed an association of improvements in BMD with decrease in fracture risk (13-15). Post hoc analyses of the FREEDOM study found that changes in BMD were inversely correlated with fracture risk (16-18). Therefore, changes in BMD over time can be considered a valid biomarker for the evaluation of the treatment effect and fracture risk reduction during treatment with denosumab agents. This study reported comparable efficacy of SB16 and DEN in comparison with previous studies using DEN as the active control. Previously reported LS mean percent changes from baseline in lumbar spine BMD at month 12 after treatment with DEN were 4.4% to 5.5% (13-15), which are comparable with the increases seen in this study with both SB16 and DEN. Secondary efficacy endpoints (ie, the percent changes from baseline in lumbar spine BMD at month 6 and the percent changes from baseline in total hip BMD and femoral neck BMD at month 6 and month 12) consistently supported the equivalence of SB16 to DEN. Moreover, these findings corroborate the efficacy of SB16 since the SABRE project demonstrated that changes in hip BMD are a considerable driver of fracture risk reduction on drug therapy (19). PD profiles were assessed by measuring the bone turnover biomarkers serum CTX and P1NP over time. Changes in serum CTX and P1NP during treatment are sensitive measures of bone turnover (20-22). Both serum CTX and P1NP profiles were similar between SB16 and DEN and consistent with observations in the FREEDOM study, showing a near-total suppression of both bone turnover markers (13). These findings further corroborate the robustness of the primary endpoint. PK profiles were comparable between SB16 and DEN at all timepoints. The safety profile of SB16 was generally consistent with that of DEN because the incidences of TEAEs, SAEs, and AESIs were comparable for the SB16 and DEN groups. There were no reports of osteonecrosis of the jaw or atypical femoral fractures in this study. The proportion of patients who experienced TEAEs leading to treatment discontinuation was comparable between the SB16 and DEN groups. However, there was a numerically higher incidence of skeletal fractures in the SB16 group compared to the DEN group during the main period. Because this study is a biosimilar study, it was not aimed at establishing efficacy per se since the efficacy of DEN in the respective therapeutic indications has already been established in the pivotal clinical trials and published literature reports. It has been shown in the FREEDOM study that the reduction of fracture risk occurs first in vertebral fractures and later nonvertebral fractures (13). All fractures observed in the main period were nonvertebral fractures and the changes in BMD and PD profiles were similar between the SB16 and DEN groups. Thus, the numerical imbalance of skeletal fractures observed in this study does not seem to be due to differences of drug efficacy.

Notably, although the incidence of hypocalcemia was comparable between the SB16 and DEN groups, an overall higher incidence of hypocalcemia was observed in this study (approximately 10%) compared to the FREEDOM study (<0.1%). This finding may be explained by a difference in the definition of hypocalcemia between the 2 studies. In the FREEDOM study, an albumin-corrected serum calcium level of <2.0 mmol/L (<8.0 mg/dL), corresponding to a CTCAE grade ≥2, was used as cutoff for hypocalcemia (13). In this study, however, the definition and reporting of hypocalcemia (as an AE) was performed at the Investigator's discretion, whereas all events of hypocalcemia were based on biochemically detected hypocalcemia; none of the cases was symptomatic. When the incidence of hypocalcemia was evaluated by the same criteria as in the FREEDOM study (albumin-corrected serum calcium level of <2.0 mmol/L [<8.0 mg/dL], corresponding to a CTCAE grade ≥2), only 4 patients (1.8%) in the SB16 group vs 5 patients (2.2%) in the DEN group fulfilled CTCAE grade ≥2 criteria for hypocalcemia in this study. It is also noted that other covariates such as estimated glomerular filtration rate, vitamin D, or PTH were similar between treatment groups, and in women with and without hypocalcemia (data not shown).

Immunogenicity was comparable between the SB16 and DEN groups. There were only 3 patients (1 in the SB16 and 2 in the DEN groups) who were nonneutralizing ADA-positive during the main period. This was consistent with previously reported immunogenicity data on treatment with DEN showing an ADA incidence <1% (5, 6).

The main strengths of the study are its robust design and adequate sample size, including total 457 randomized patients representative of the intended patient population, to support the validity of the efficacy comparison between SB16 and DEN. However, because this study was initiated and designed to assess equivalence of a biosimilar to its reference product, a larger sample size to determine long-term fracture risk was not considered, which could be a potential limitation of this study.

## Conclusions

The presented efficacy, safety, PK, PD, and immunogenicity data in patients with PMO further support the biosimilarity of SB16 to DEN from a totality-of-the-evidence perspective. SB16 was shown to be equivalent to DEN in terms of percent change from baseline in lumbar spine BMD at month 12 in patients with PMO. Other efficacy endpoints including changes in hip BMD, PK, PD, and safety, and immunogenicity profile were comparable between the SB16 and DEN groups.

## Data Availability

Upon request, and subject to certain criteria, conditions, and exceptions, Samsung Bioepis will provide access to individual deidentified participant data to researchers whose proposals meet the research criteria and other conditions and for which an exception does not apply. Proposals should be directed to the corresponding author. For access, data requestors must enter into a data access agreement with Samsung Bioepis.

## Abbreviations

ADA: antidrug antibodies
AEs: adverse events
AESIs: adverse events of special interest
CTCAE: Common Terminology Criteria for Adverse Events
BMD: bone mineral density
CTX: C-telopeptide of type I collagen
DEN: denosumab
FAS: Full Analysis Set
LS: least-squares
PD: pharmacodynamic
PK: pharmacokinetic
PMO: postmenopausal osteoporosis
P1NP: procollagen type I N-terminal propeptide
PPS: per-protocol set
SAEs: serious adverse events
SAF1: Safety Set 1
TEAE: treatment-emergent adverse event
